# A spliced form of CD44 expresses the unique glycan that is recognized by the prostate cancer specific antibody F77

**DOI:** 10.18632/oncotarget.23341

**Published:** 2017-12-16

**Authors:** Xi Chen, Yasuhiro Nagai, Zhiqiang Zhu, Hang Ruan, Donna M. Peehl, Mark I. Greene, Hongtao Zhang

**Affiliations:** ^1^ Department of Pathology and Laboratory Medicine, Perelman School of Medicine, University of Pennsylvania, Philadelphia, PA 19104, USA; ^2^ Department of Urology, Stanford University School of Medicine, Stanford, CA 94305, USA

**Keywords:** prostate cancer, antibody, post-translational modification, O-linked glycosylation, CD44

## Abstract

Prostate cancer is the most common cancer occurring in men in the United States. The monoclonal antibody F77 that was originally developed in our laboratory recognizes mainly glycolipids as well as O-linked glycosylation on proteins in prostate cancer cells. We have identified a spliced form of glycoprotein CD44 as one critical protein expressing the F77 antigen. The F77-specific glycosylation occurs on multiple potential glycosylation sites on the CD44 protein encoded by the fourteenth exon. CD44 is a tumor stem cell marker and is known to induce tumor stemness and metastasis. Knockdown of CD44 or FUT1 genes dramatically reduced F77-induced apoptosis in prostate cancer cell lines. We developed an ELISA using both a CD44 antibody and F77 to identify the special form of glycosylated CD44 from prostate cancer cells as well as from serum samples of prostate cancer patients. These results reveal a CD44-dependent mechanism for F77 to induce tumor cell apoptosis, and a new strategy for the detection of glycosylated CD44 proteins secreted by prostate cancer cells.

## INTRODUCTION

Prostate cancer is the most common cancer in men and one of the leading causes of cancer-related death in the United States [1]. Although localized prostate cancer is often cured, patients with distant metastases have a 5-year survival rate of 28.4%. New approaches for the treatment of advanced and metastatic prostate cancer are needed. In addition, although serum prostate-specific antigen (PSA) has been used as a biomarker to screen for prostate cancer, measurement issues lead to over-diagnosis, resulting in overtreatment of prostate lesions that do not necessarily require therapy [2]. Therefore, alternative or additional methods are needed for accurate diagnosis and prognosis of prostate cancer to determine whether medical treatment is needed.

Antibody-based therapeutics have evolved for cancer therapy [3]. In particular, tumor cell-specific antibodies such as anti-CD20 (rituximab) and anti-erbB2/neu (trastuzumab) antibodies have proven to be effective in clinical cancer treatment [4, 5]. These targeted therapeutic antibodies not only have inhibitory effects on tumor cells by binding to targeted molecules, but also lead to heightened host immunity against tumors [6]. Recently, we demonstrated that anti-erbB2/neu targeted antibody can promote the development of tumor specific CD8+ T cells in a syngeneic MMTV-neu mouse model [7].

There are several prostate cancer-specific antigens that can serve as targets for antibody therapy [8]. Prostate-specific membrane antigen (PSMA) is highly expressed in prostate cancer cells and increased expression correlates with advanced disease and metastasis [9]. Accordingly, PSMA is being actively exploited as a possible target for treatment of prostate cancer [10]. Prostate stem-cell antigen (PSCA), a glycosylphosphatidylinositol (GPI)-anchored protein overexpressed in prostate cancer cells [11], has also been exploited as a possible target for immunotherapy. Monoclonal antibodies against PSCA showed significant inhibition of growth of androgen-dependent tumor xenografts [12]. However, targeting PSCA was less effective in clinical trials [8]. In addition, anti-PSCA antibodies are ineffective against androgen-independent tumors, since PSCA is minimally expressed in such cells [12].

We have developed prostate cancer-specific monoclonal antibody (mAb) named F77 by immunizing mice with the androgen-independent prostate cancer cell line PC3 [13]. Immunohistological analysis showed that this antibody stained 112 of 116 primary and 29 of 34 metastatic human prostate cancer specimens with marked specificity for prostate tumor cells [14]. Some staining of breast tumors [13] and cold agglutinin type binding to erythrocyte antigens has been observed [15].

F77 can inhibit the *in vivo* growth of PC3 tumors and other androgen-independent prostate tumors such as DU145 [14]. F77 recognizes protein O-glycan modifications catalyzed by glycosyltransferases such as fucosyltransferase 1 (FUT1) and glutaminyl (*N*-acetyl) transferase (GCNT)1–3 [15, 16]. In this study, we sought to identify glycosylated proteins recognized by F77 as well as the possible glycosylation sites on the protein, and assessed the potential use of the F77 glycoprotein antigen as a novel diagnostic biomarker for prostate cancer.

## RESULTS

### CD44 expresses the antigen for mAb F77

By immunoprecipitation and mass spectrometry, previous studies identified CD13, CD44, Na^+^/K^+^ ATPase, and cytoskeleton proteins as proteins that expressed the antigen for the prostate cancer-specific mAb F77 [14]. The present studies have focused on CD44, a transmembrane glycoprotein that is important for cell signaling and tumor stemness and metastasis [17, 18].

**Figure 1:**
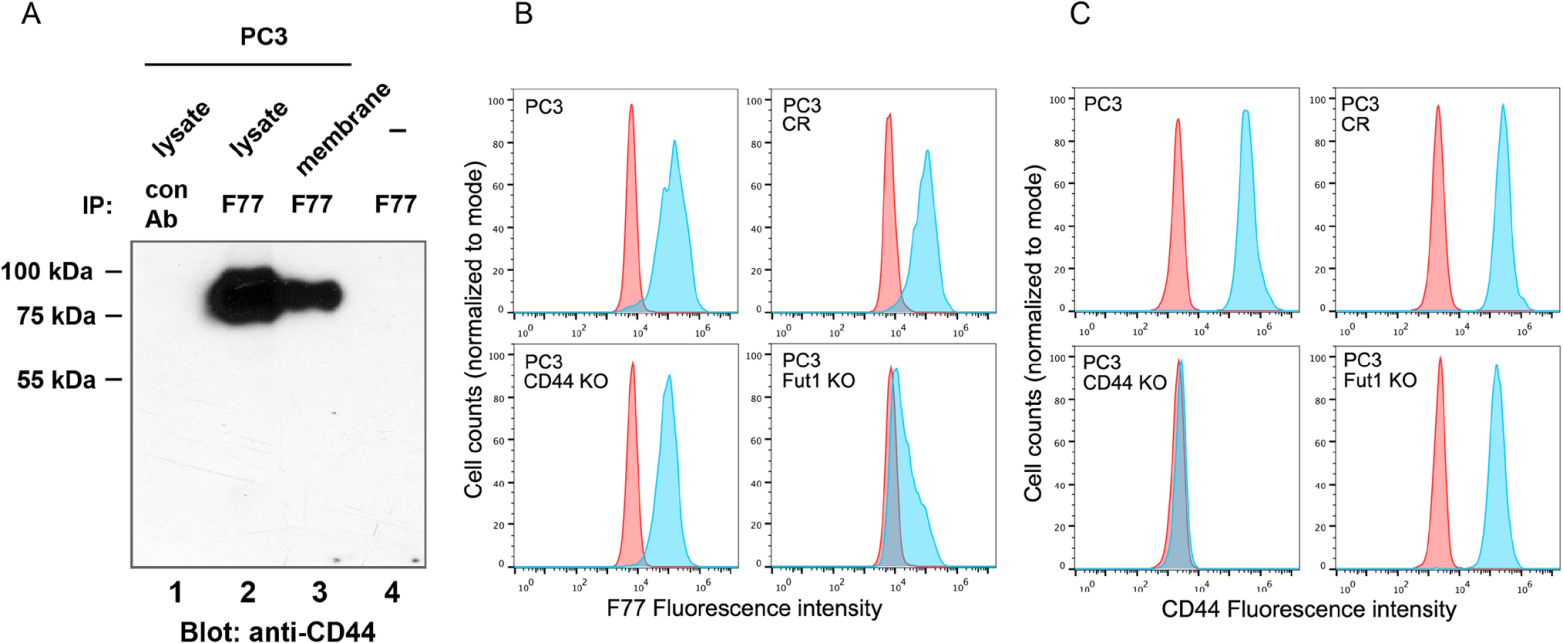
Identification of CD44 as one glyco-protein recognized by mAb F77 (**A**) CD44 was immunoprecipitated by mAb F77 from PC3 cell whole lysate and membrane fractions. (**B**, **C**) F77 (B) and CD44 (C) FACS staining on PC3, CRISPR control (PC3 CR), CD44 knockout (PC3 CD44 KO), and FUT1 knockout (PC3 FUT1 KO) cell lines. Blue peaks represent the staining by specific antibodies, while red peaks represent the control staining.

In the prostate cancer cell line PC3, an isoform of CD44 with the molecular weight of 85–90 kDa was predominantly precipitated by mAb F77 (Figure 1A and Supplementary Figure 1). This CD44 species exists in both cytoplasmic and membrane fractions. We employed CRISPR technology to specifically knock-out (KO) CD44. As shown in Figure 1B and 1C, CD44 KO only modestly reduced F77 staining in PC3 cells (a reduction of 15%) (Figure 1B), indicating that CD44 is one of many proteins or glycolipids that are targets of F77-specific glycosylation.

FUT1 and one of GCNT1, GCNT2 or GCNT 3 are essential enzymes for F77-specific O-linked glycosylation in prostate cancer cells [16]. As expected, knockout of FUT1 dramatically decreased F77 binding activity (Figure 1B). Interestingly, FUT1 KO also moderately reduced CD44 expression in PC3 cells (35–50%), suggesting that FUT1 modifications are necessary for CD44 expression (Figure 1C).

We investigated whether the F77 antigen and CD44 were co-localized in PC3 cells. As shown in a confocal imaging study, F77 staining co-localized with anti-CD44 signals (Supplementary Figure 2), and pre-incubation of F77 with PC3 cells induced the formation of clusters in PC3 membranes (Supplementary Figure 2, white arrows).

### Identification of the F77-epitope region in CD44

One of CD44’s functions is to bind to hyaluronic acid. Previous studies have shown that blocking hyaluronic acid binding to CD44 induces apoptosis [19–21]. To investigate if F77 prevents CD44 binding to hyaluronic acid, we performed competitive binding assays using FITC-labeled hyaluronic acid and F77 mAb. In the presence of F77, binding of FITC-labeled hyaluronic acid to CD44 was not significantly diminished (Supplementary Figure 3A). In addition, high concentrations of hyaluronic acid did not inhibit PE-labeled F77 binding (Supplementary Figure 3B, 3C), indicating that F77 does not block CD44 from binding to hyaluronic acid. We speculated that F77 binding to CD44 occurred at a distinct region from the hyaluronic acid binding site.

**Figure 2:**
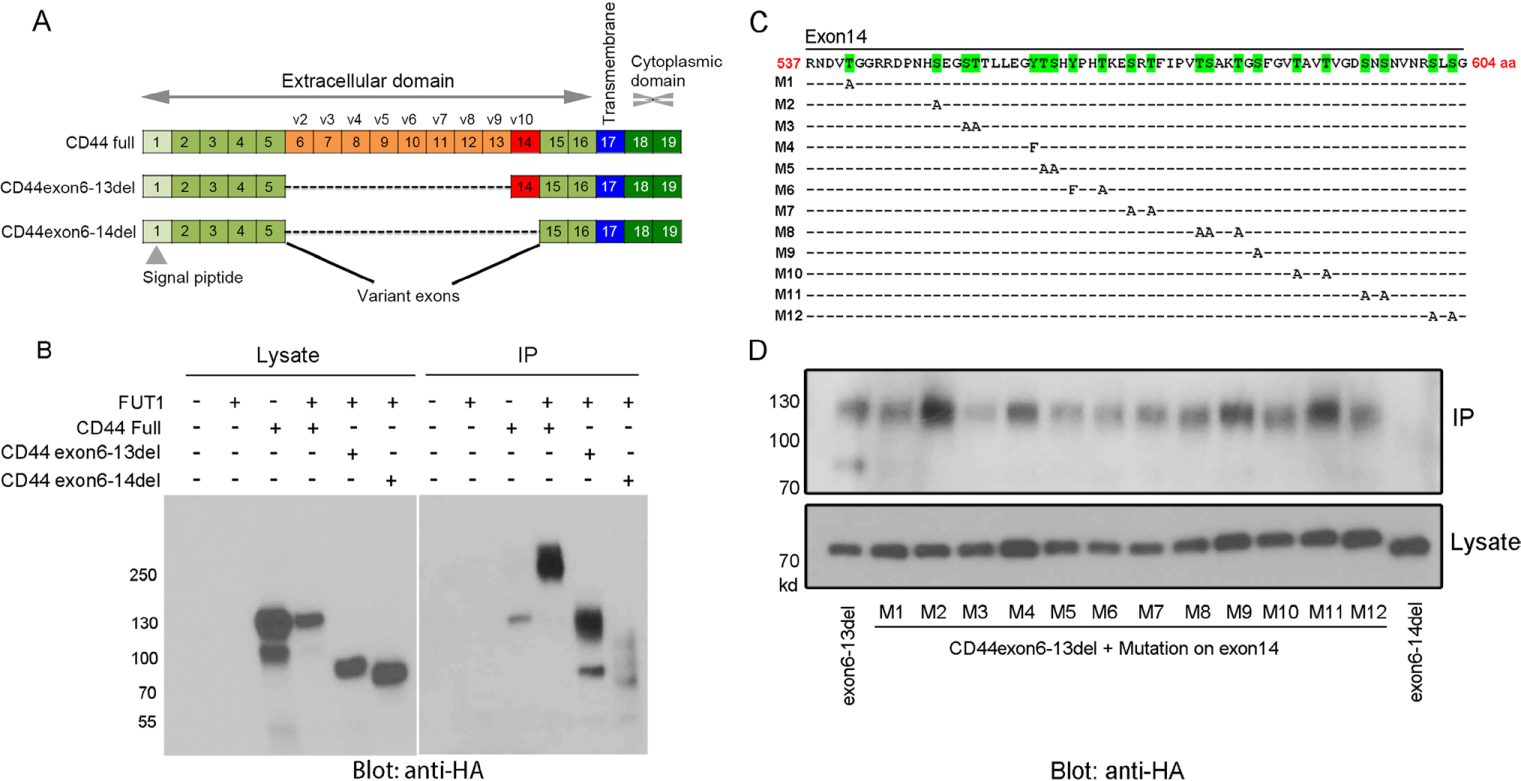
Mapping F77 binding epitope region and potential glycosylation sites on CD44 (**A**, **C**) Schematic diagram of CD44 gene structure and exon deletion, as well as point mutation of potential glycosylation sites on exon 14 of CD44 (CD44v10). Point mutation constructs (C) were generated from the pIPHA2-CD44exon6-13del plasmid (A). (**B**, **D**) F77 immunoprecipitation of ectopically expressed isoforms of CD44 and various point mutations at potential glycosylation sites in exon 14 of the CD44v10 isoform. (B) 293T cells were transfected with the pIPHA2 vector carrying different fragments of CD44 with or without FUT1 expression plasmid. (D) 293T cells were transfected with FUT1 and pIPHA2-CD44exon6-13del plasmid with different O-glycosylation amino acid mutations on exon 14. FUT1 and pIPHA2-CD44exon6-13del were used as the positive control, while FUT1 and pIPHA2-CD44exon6-14del worked as the negative control. The lower panel in (D) showed the loading control from cell lysates. Cell lysates and F77-precipitated proteins were examined by western blotting using anti-HA-HRP.

CD44 protein has different isoforms ranging from 85 to 230 kDa. We evaluated which isoform and region of glycosylated CD44 mediates F77 binding. Among the 10 CD44 variant exons (v1-v10), exon v1 protein only exists in mouse but not in human, due to a stop codon in the human gene [17]. We annotated the human CD44 exons according to the various isoforms [22], and constructed pIPHA2 vectors carrying different species of CD44 to co-express with FUT1 in 293T cells (Figure 2A): full length, fragments with deletion of exons 6–13 or 6–14 (Figure 2A, 2B). In the absence of ectopically expressed FUT1, full length CD44 was expressed as a 130-kDa band and could only be weakly precipitated by F77. This can be explained by the fact that 293T cells express minimal amounts of endogenous FUT1 [16]. In contrast, in the presence of FUT1, although 130-kDa CD44 remained the dominant band in the lysate, F77 preferentially immunoprecipitated a high molecular weight form of CD44 (∼250-kDa). For the CD44 construct with exons 6–13 deleted, F77 was able to precipitate the dominant 75-kDa band from the lysate, as well as the highly glycosylated form of ∼130 kDa. The sizes of both bands were smaller than the corresponding bands from the full length construct. The CD44 construct with the deletion of exons 6–14 expressed a dominant 65-kDa band in the lysate, but F77 only very weakly immunoprecipitated this band and failed to precipitate a highly glycosylated band (Figure 2B). These data indicate that exon 14 of CD44 is important for F77-specific glycosylation.

We further studied potential glycosylation sites in exon 14. Using the pIPHA2-CD44exon6-13del construct, we mutated every single potential glycosylation site in exon 14: Serine (S) and Threonine (T) were mutated to Alanine (A), and Tyrosine (Y) was mutated to Phenylalanine (F) (Figure 2C). Mutation of most of these sites, except those on constructs M2, M4, M9, and M11, resulted in decreased F77 immunoprecipitation of CD44 (Figure 2D), indicating that the F77-specific glycosyltransferase modification occurs on various sites in exon 14. Our studies revealed that a short sequence of about 21 amino acids from T561 toT581 was critical for glycosylation. When amino acid residues T561, S562, T577, S578 and T581 were simultaneously mutated to alanine, the density of the F77-precipitated CD44 band was diminished by ∼ 80% (Supplementary Figure 4).

It was interesting to notice that several mutations, especially the M2 variant of CD44, appeared to lead to increased F77 signals (Figure 2D). In the study of a herpes simplex virus type 1 (HSV-1) glycoprotein, replacing basic residues around the O-linked glycosylation site also increased glycosylation [23]. *In vitro* assay confirmed that these mutations increased glycosylation efficiency. In our study, the replaced amino acid residues were Ser, not basic residues, but an analysis of the O-glycosylation sites revealed that the Ala residue is preferred around O-glycosylated Ser/Thr, especially in the -10, -4, -2, -1, and +2 positions [24]. Clearly, Ser at these positions were unlikely glycosylation sites, but they might be close to the real ones.

### Knockout of CD44 or FUT1 in PC3 limits F77-induced apoptosis

**Figure 3:**
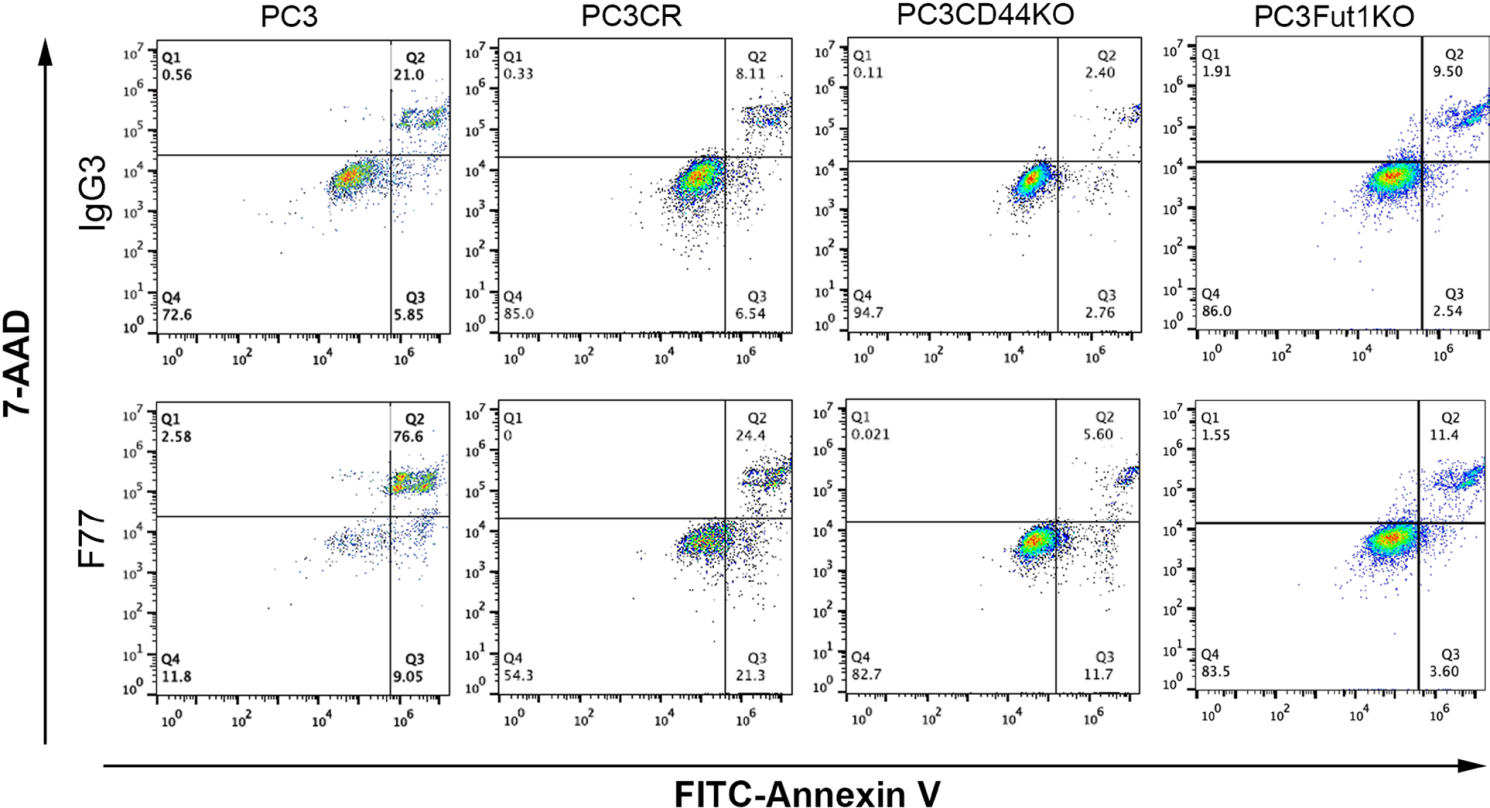
FACS analysis of apoptosis in CRISPR cell lines derived from PC3 PC3, CRISPR control (CR), CD44 KO and FUT1 KO cells were treated with mAb F77 or the control mouse IgG3 antibody, and analyzed by FITC-labeled Annexin V and 7-AAD. F77 treatment resulted in 85.6% and 45.7% (Q2 + Q3) of apoptosis/necrosis (Annexin V positive) in PC3 and PC3CR cells at 4°C, respectively, while CD44 KO and FUT1 KO cells showed much reduced rates at 17.3% and 15.0% (Q2 plus Q3), respectively.

Previously, we found that F77 could induce modest apoptosis in PC3 cells [14]. In a study using glycolipid microarrays, F77 appeared to have higher affinity for its antigen at low temperatures (4°C), which is in accord with the cold agglutinin behavior of this antibody [15]. We evaluated the ability of F77 to induce apoptosis at different temperatures. As expected, we noticed that F77 induced much more dramatic apoptosis at 4°C than at 37°C, with greater than 80% of cell death in PC3 cells (Q2 and Q3, Figure 3). This elevated apoptosis at the low temperature was also observed in the tumorigenic and F77-positive prostate epithelial cell line RWPE-2, which was derived from RWPE-1 human epithelial cells transformed by the *K-ras* oncogene. We did not detect apoptosis in the parental F77-negative RWPE-1 cell line, indicating the effect was F77-binding dependent (Supplementary Figure 5).

We generated CD44 or FUT1 CRISPR knockout PC3 cells (Figure 1B), and then tested F77- induced apoptosis in these lines. As shown in Figure 3, elimination of CD44 or FUT1 greatly limited F77 mAb-induced apoptosis at low temperature. While 45% apoptosis was observed in the control cell line PC3_CR, only about 15% cell death was detected in FUT1 or CD44 KO cell lines. CD44 has been shown to promote resistance to apoptosis in some cancer cells [25]. These data further confirm that F77-induced apoptosis is highly dependent on glycosylation mediated by FUT1 and that CD44 is a main carrier protein for F77-specific glycosylation.

**Figure 4:**
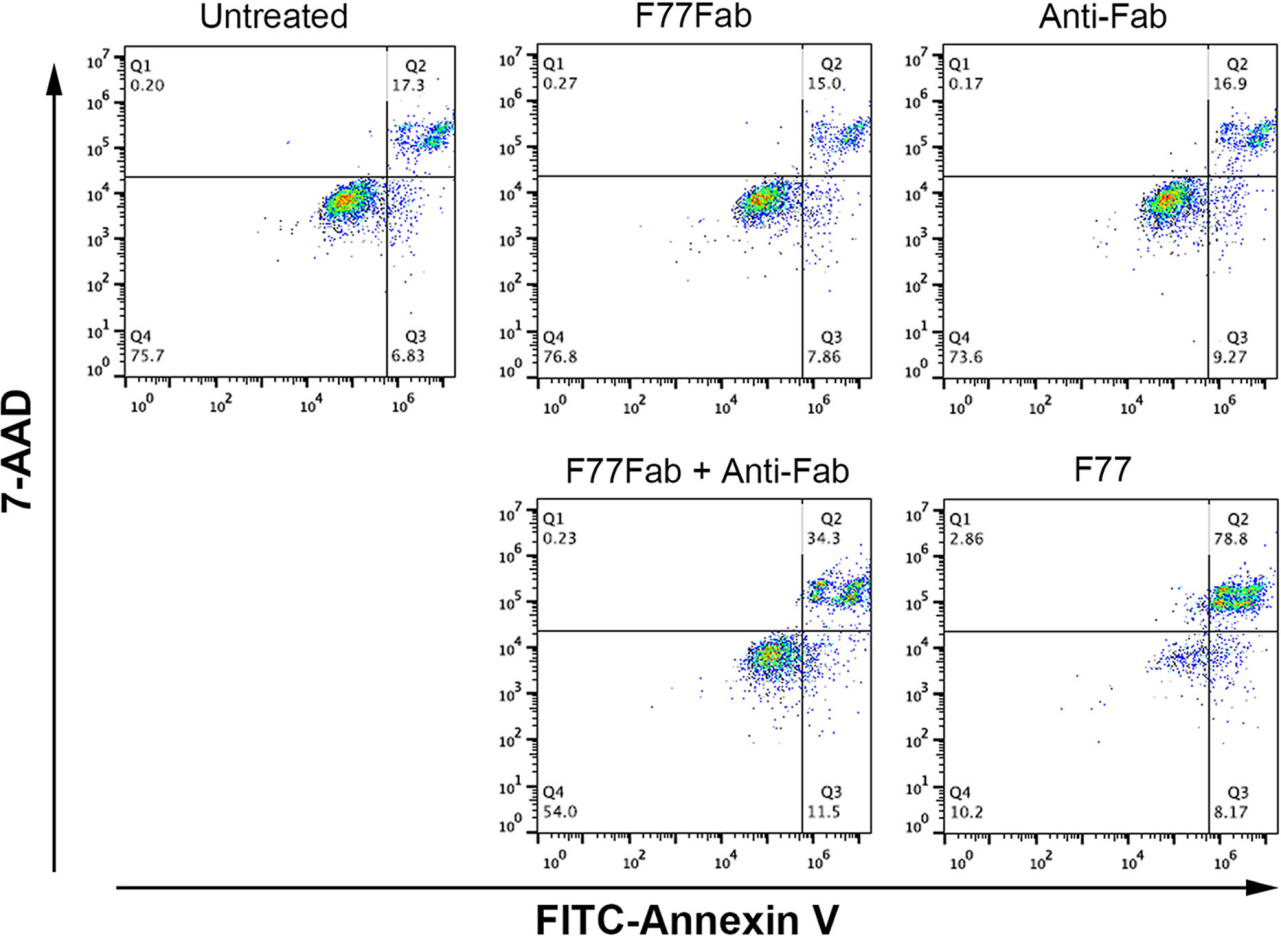
mAb F77-induced cell death in PC3 cells is dependent on bivalency PC3 cells were treated with 10 μg/mL of the monovalent Fab fragment (F77Fab), anti-Fab antibody control, F77Fab plus anti-Fab (bivalent) or intact mAb F77 (bivalent) on ice for 30 min. After antibody treatment, cells were washed and stained with FITC Annexin V and 7-AAD at room temperature for 15 min before FACS analysis. F77Fab or anti-Fab antibody control treatment resulted in similar apoptotic signals as the untreated control. Bivalent assembled F77Fab and anti-Fab complex partially rescued the ability of F77Fab to induce cell death, showing more Annexin V-positive staining than F77Fab or anti-Fab alone.

We further examined whether the monovalent F77 Fab fragment could also induce cell death *in vitro*. The Fab species was prepared by pepsin digestion followed by sieve preparative chromatography. It was observed that the monovalent Fab was only able to induce very limited cell death. However, in the presence of a goat anti-mouse Fab antibody, which crosslinks Fab into a bivalent binding unit, significant cell death was observed again (Figure 4). These data suggest that bivalency of F77 mAb is required for induction of apoptosis in the tumor cells.

### A novel ELISA to detect F77-glycosylated CD44 in prostate cancer cell culture media and sera from prostate cancer patients

**Figure 5:**
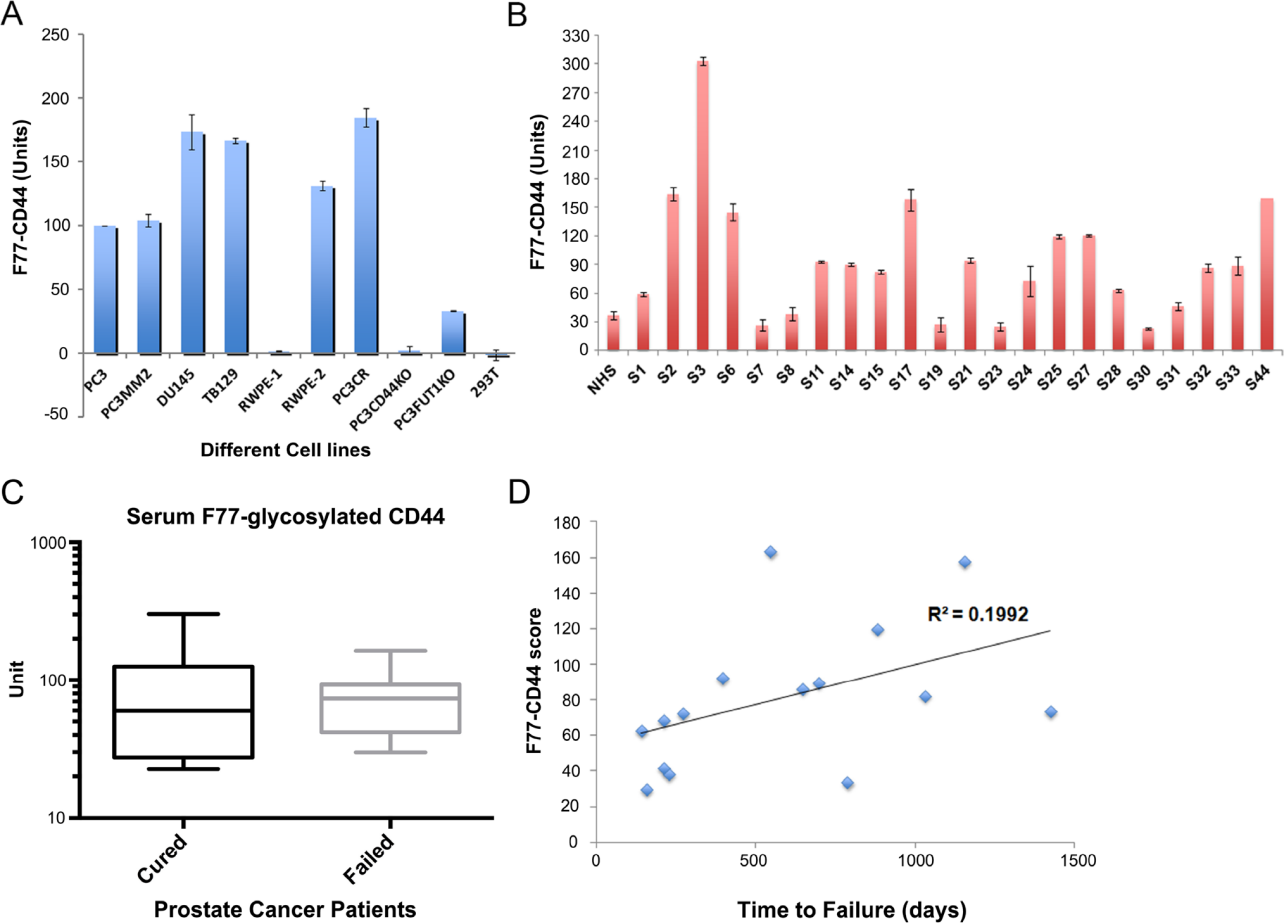
Detection of F77-glycosylated CD44 (F77-CD44) by ELISA (**A**) Verification of the F77-CD44 ELISA using various cell lines. F77 served as the capture antibody and biotinylated anti-CD44 (clone IM-7) was used as the detection antibody. Cell culture supernatants obtained from cell lines as indicated were used as the samples. (**B**) Examination of sera from prostate cancer patients. NHS: normal healthy serum. S1–S44: Prostate cancer patients’ samples. (**C**) No difference in the serum F77-CD44 levels between cured patients and patients with biochemical failure after prostatectomy. Unpaired t test was used to compare the levels between these two groups of samples. *P* = 0.79. (**D**) Correlation analysis between F77-CD44 ELISA results and the PSA failure status of the patients. The X axis shows the time for patients to show detectable PSA failure after surgery. The Y axis shows the F77-CD44 score.

Since CD44 is a target of F77-related glycosylation, we next investigated whether this glycosylated form of CD44 could be detected as a secreted protein in the culture media of prostate cancer cells, or in sera from men with prostate cancer. A sandwich ELISA was designed in which F77 was used as the capture antibody and an anti-CD44 antibody (clone IM-7) was used as the detection antibody. Using this assay, F77- glycosylated CD44 was detected in the media from 3 prostate cell lines (PC3, PC3-MM2 and DU145) (Figure 5A). Culture medium from the normal prostate cell line RWPE-1 was negative, but culture medium from the *K-ras* transformed clone RWPE-2 was positive. The result is consistent with the mAb F77 staining on these cells by FACS [14]. Of note, medium in which the breast cancer cell line TB129 was cultured was also positive in the ELISA. This is also consistent with the observation of some levels of F77 staining in certain breast cancer cell lines and tissues [26]. As expected, the ELISA signal was reduced in the FUT1 KO PC3 line and not detected in the CD44 KO line and 293T cells.

F77-glycosylated CD44 was also detected in pre-prostatectomy sera from 22 men with Gleason score 7 prostate cancer (Figure 5B). Of the 22 sera, 18 had higher levels of F77-glycosylated CD44 compared to normal healthy controls. Ten of 22 men had biochemical (PSA) recurrence following radical prostatectomy. Levels of F77-glycosylated CD44 were not significantly different in the sera of the 10 men who failed to be cured by radical prostatectomy compared to the 12 men who were cured by surgery (Figure 5C). However, a correlation of F77-glycosylated CD44 values and time to biochemical recurrence in the sera of the 10 men who failed to be cured was noted (Figure 5D).

## DISCUSSION

Previous studies from our lab have shown that the prostate cancer-specific mAb F77 is potentially useful for therapy of prostate cancer [13, 14]. Based on our previous reports, F77 recognizes glycolipids as well as fucosylated O-glycan proteins [14–16]. In the present study, we found that FUT1 expression is essential for the presentation of F77 antigens. We also confirmed CD44 as a target glycosylated protein for F77 in prostate cancer cells (Figure 1), and we further mapped the critical F77 binding region to exon 14 (Figure 2). Through a point mutation strategy, we are able to narrow glycosylation patterns relevant to F77 binding to a 21- amino acid region within exon 14, which contains potential glycosylation sites for FUT1- mediated modification (Figures 2 and Supplementary Figure 3).

CD44 is a single pass trans-membrane glycoprotein involved in cell-cell and cell-matrix communication. It is also known that CD44 is highly expressed in cancer stem cells, which are highly resistant to apoptosis and are thought to be essential for metastasis [18]. CD44 consists of twenty exons and has different isoforms with molecular weights ranging from 85 to 230 kDa due to alternative splicing, post-translational modification and partial cleavage [27]. CD44 standard (CD44s) is the shortest form of CD44 and is present in most vertebrate cells. In contrast, the splice variant isoform of CD44 (CD44v) is only expressed in some epithelial cells during embryonic development, in lymphocytes during maturation and activation, and in several types of carcinoma [28, 29]. Some CD44v species in tumors are associated with *K-*ras-mediated transformation [29] and represent attractive targets for immunotherapy. Several therapeutic strategies targeting CD44 have led to clinical trials [18], but none seems to lead to successful therapies at this time point [30, 31]. As shown in our previous study [14], F77 is highly specific to prostate cancer, and the current study indicates that F77 recognizes a special glycosylated form of CD44, CD44v10 at the exon 14 region (Figure 2).

Our study also showed that bivalent F77 can induce apoptosis of prostate cancer cells, especially at 4°C (Figure 3). The increased activity at low temperature correlates with the higher binding affinity of F77 to its antigen at 4°C [15]. Meanwhile, CD44 deletion almost completely abrogated F77 –induced apoptosis (Figure 3). An anti-CD44 mAb A3D8, which binds to CD44 at the non- hyaluronic acid binding region, has also been observed to induce apoptosis in acute myeloid leukemia cells by inducing lipid raft clustering [32].

We detected F77-glycosylated CD44 in the sera of men diagnosed with primary prostate cancer. All of these men had Gleason score 7 cancer and were treated by radical prostatectomy. While levels of F77-glycosylated CD44 were higher in the majority of men with cancer compared to the value of serum from healthy controls, our study only involved a small number of clinical samples. Similarly, levels of F77-glycosylated CD44 did not predict progression (biochemical recurrence) following surgery in this small study, although there was a correlation between higher levels and longer time to failure. Since our data suggest that CD44v10 may be most relevant to F77 specific glycosylation in prostate cancer, future studies using an antibody specific to this form of CD44 may yield more relevant data than our current ELISA.

In conclusion, we have discovered CD44 as a major protein carrying the F77 epitope at the exon 14 region. F77 can induce apoptosis on prostate tumor cell lines in a bivalent- and CD44-dependent manner. Our study also suggests that serum levels of F77-glycosylated CD44 may have potential utility to facilitate the diagnosis or prognosis of prostate cancer. Since we have identified the specific region that harbors the glycosylation sites, a much more specific diagnostic assay can be developed to monitor the special glycosylated form of CD44 during prostate cancer progression.

## MATERIALS AND METHODS

### Cells and antibodies

Prostate tumor cell lines were cultured as described previously [14]. The F77 antibody was purified in our laboratory [13]. All cell lines were routinely checked for mycoplasma. PC3 cell lines were confirmed virus free by the IMPACT II test (IDEXX Bioresearch). Anti-CD44 (IM-7), biotinylated anti-CD44 (IM-7) and anti-mouse IgG were purchased from Biolegend. Anti-mouse IgG FITC was purchased from Jackson ImmunoResearch. Anti-rat AF594 was purchased from Invitrogen.

### Flow cytometry assays

Cells were incubated with the anti-CD44 PE or the F77 antibody for 20 min on ice. Cells were then washed with FACS buffer (0.5% BSA/PBS), then incubated with anti-mouse IgG AF488 for 20 min at 4°C to detect F77. After washing and collection, the cells were examined on an Accuri C6 flow cytometry (BD Bioiscience) and analyzed using Flowjo software.

### Plasmids and CRISPR-Cas9 gene knock out

All CRISPR-CAS9 constructs were purchased from Genecopoeia. CD44 expression vector was obtained from GE Life Sciences, then subcloned into pIPHA2 vector as described previously [33]. FUT1 expression vector was provided by Dr. Minoru Fukuda [16]. Knockout of targeted genes in PC3 cells was performed according to manufacture’s instructions. In brief, PC3 cells were seeded in 6-well plates at 3 × 10^5^ cells/well the night before transfection. Cells were then transfected with 2 μg/well of the all-in-one CRISPR-CAS9 vectors using Fugene6 (Roche) according to manufacturer’s instructions. After 24 hr, media were changed and cells were incubated with 600 μg/ml of G418 for 24 hr, then media were changed to normal growth media and cells were cultured until stable growth was observed. The cells were then sorted and collected as CD44-negative or F77-low (for FUT1) fractions and termed as knocked down cells using FACS Aria (BD Bioscience). For the FUT1 deletion, genomic PCR was used as a further validation of FUT1 gene deletion and confirmed as the genomic deletions of targeted sequences. For the exon deletion and point mutations of CD44, the vectors were constructed using the QuikChange Site- Directed Mutagenesis Kit (Stratagene) and verified by sequencing. The primers used in this work are listed in Supplementary Table 1.

### Immunoprecipitation and western blotting

PC3 cells were lysed with low salt lysis buffer [20 mM Tris-Cl pH 7.5, 150 mM NaCl, 1% TritonX-100, and complete mini protease inhibitor cocktail (Roche)] and subjected to immunoprecipitation. Five micro gram of F77 antibody were bound to Dynabead protein G magnetic beads (Invitrogen) according to manufacturer’s instructions, then incubated with the lysate for 2 hr. For membrane proteins, we first incubated mAb F77 (5 μg) with PC3 cells, then lysed cells with low salt lysis buffer and precipitated with protein G magnetic beads. The precipitates were then washed three times with the buffer (20 mM Tris-Cl pH 7.5, 150 mM NaCl, 0.01% Tween-20) and boiled for 5 minutes in SDS loading buffer. Samples were analyzed by SDS-PAGE, transferred to Immobilon-P (Millipore) PVDF membranes, and probed using anti-CD44 antibody, and detected with anti-rat IgG HRP (Santa Cruz Biotechnology) and Immobilon Western Chemiluminescent horseradish peroxidase (HRP) Substrate (Millipore). For glycosylation site analysis, 293T cells were transfected with FUT1 and CD44 mutant plasmids using Fugene6. The cells were then lysed with low salt lysis buffer and subjected to SDS PAGE and western blotting as described above. HA-tagged proteins were detected with anti-HA Peroxidase (3F10; Roche) using ECL.

### Apoptosis assay

Cells were detached and incubated with either 10 μg/mL mAb F77 or the control antibody mouse IgG3 for 30 min at 4°C or 37°C. After washing with FACS buffer, cells were stained with 5 μL FITC-Annexin V or 7-AAD (Biolegend) according to manufacturer’s instructions. Positive controls were established by heat-shocking PC3 cells at 56°C for 20 min before staining with FITC-Annexin V or 7-AAD. Samples were analyzed by BD Accuri C6 flow cytometry (BD Biosciences) and Flowjo software.

### ELISA assay

High binding 96-well ELISA plates (ISC T3021–4) were coated with 100 mL mAb F77 for a final concentration of 5 ug/mL, and incubated overnight at 4°C. The next day, the plate was washed three times with 200 mL PBS-T (PBS containing 0.05% Tween-20), and incubated with 300 mL blocking buffer (1x Casein in PBS, SurModics, BioFX Phosphate Buffered Saline/Casein Block and Diluent) for 1 hr at room temperature. The plate was washed again, and 50 mL of cell culture medium or serum were added for 1 hr incubation at RT. After washing, biotin anti-CD44 was diluted 1:1000 (stock is 0.5 mg/mL) in PBS, and 100 mL were added to the wells and incubated 1 hr at RT. After washing, SA-HRP (R&D) was diluted 1:200, and 100 mL were added to the wells for incubation at RT for 1 hr. The substrate solution was prepared by dissolving 1 capsule of Phosphate-Citrate Buffer with Sodium Perborate Capsules (Sigma), in 50 ml of water to yield 2X solution. Five mL of the 2X solution was mixed with 5 mL of water to make phosphate-citrate buffer (0.05M, pH 5.0). One tablet of 3,3, 5,5′-Tetramethylbenzidine dihydrochloride (TMB) (Sigma) was dissolved in the dark in the phosphate-citrate solution. The plate was washed six times, and 100 mL developer substrate solution was added to each well and the plate was incubated at RT in the dark for 30 min. Finally, 50 mL of 2 M H_2_SO_4_ was added to stop the reaction. The plate was read at 450 nm with the reference wave length at 620 nm. A batch of PC3 cell culture medium, from confluent cells, was collected and used to define the standard F77-CD44 unit for future study. The reading of undiluted medium for this batch of PC3 cells was defined as 100 units. This standard medium batch was aliquoted and stored at –80°C.

### Confocal microscopy

PC3 cells (50,000/well) were seeded into 8 well Lab-Tek chamber slides (Corning) and cultured for 2 days. The cells were washed once with FACS buffer, and then treated with a gradient of 0, 1, and 5 μg/ml of F77 for 30 min at 4°C. After washing 3 times with the FACS buffer, cells were fixed with 4% paraformaldehyde/PBS for 10 min at 4°C. After washing 3 times, cells were incubated with the blocking buffer (3% normal goat serum/PBS) for 1 hr at RT, then incubated with anti-CD44 antibody (1:250) for 2 hr at RT. Cells were then washed three times with the FACS buffer and then incubated with anti-mouse-FITC IgG (1:200 dilution) and Alexa Fluor 594 anti-rat IgG (1:400 dilution). After washing, the chamber was removed and coverslips were loaded using VECTASHIELD mounting medium (Vector Laboratory). The stained cells were examined with a Leica STED 3X Super-Resolution Microscope.

### Serum samples

Normal human sera (NHS) was a pooled sample from healthy donors [34]. Sera from 22 men who had undergone radical prostatectomy at Stanford University between 1993 and 2003 to treat prostate cancer were used to measure F77-glycosylated CD44. All patients were consented with Institutional Review Board-approved protocols. Pre-prostatectomy blood was drawn in tubes with silica clot activator. Following centrifugation, the sera were collected, aliquoted, and stored at –80°C until analyzed. Patient characteristics and histopathological/morphological variables were obtained from an existing database.

Biochemical failure, also called PSA failure, is used here as a longitudinal outcome measure. Biochemical failure is defined as two consecutive PSA values above a cutoff point of 0.07 ng/ml (for PSA measured by the Tosoh method) or 0.2 ng/ml (for measurements by less sensitive methods). When a subject experienced PSA failure, time to failure was calculated as the number of days between the date of surgery and the first of the two consecutive PSA values that exceeded the cutoff point.

## SUPPLEMENTARY MATERIALS FIGURES AND TABLE


