# Supplementary material for: A spliced form of CD44 expresses the unique glycan that is recognized by the prostate cancer specific antibody F77
Source: Oncotarget. 2017 Dec 16;9(3):3631–40. doi: 10.18632/oncotarget.23341 (PMC5790488; doi:10.18632/oncotarget.23341)
Supplement: Supplementary file 1 [file oncotarget-09-3631-s001.pdf]

A spliced form of CD44 expresses the unique glycan that is recognized by the prostate cancer specific antibody F77

SUPPLEMENTARY MATERIALS

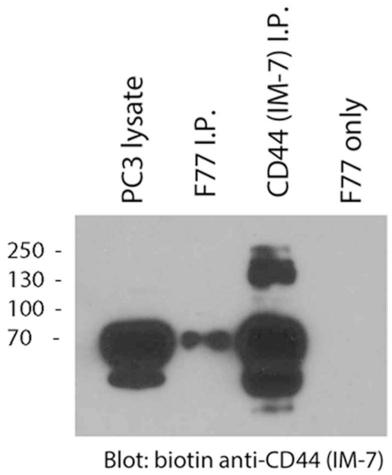

Supplementary Figure 1: CD44 expression in PC3 cells and the co-immunoprecipitation by F77. PC3 cell lysate was used for mAb F77 and anti-CD44 (clone: IM-7) immunoprecipitation. Anti-CD44 clone IM-7 was reported to recognize an epitope located in exon 5, between amino acids 145 and 186, which is shared by all isoforms of CD44. Biotin anti-CD44 (IM-7) and streptavidin-HRP were used for western blotting detection.

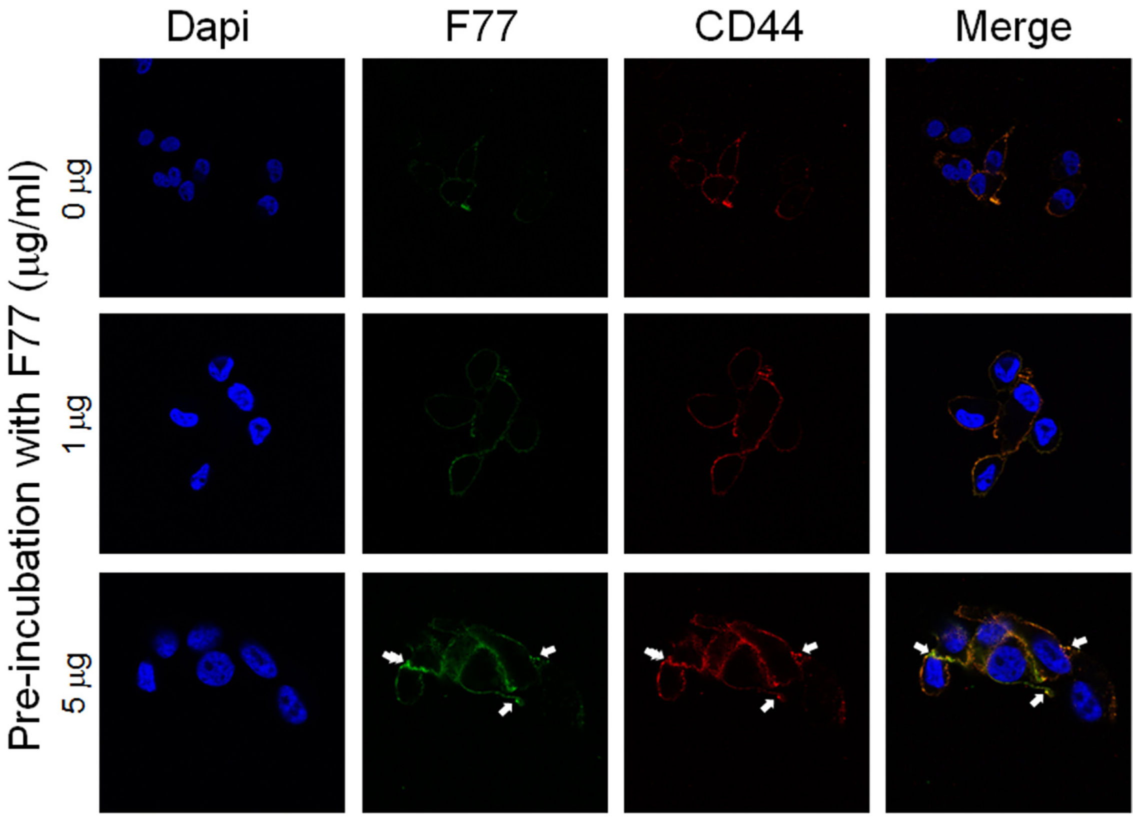

Supplementary Figure 2: Co-localization of F77 and CD44 in F77-treated PC3 cells. PC3 cells were incubated with indicated concentrations of F77 for 30 min at 4°C. After washing, the cells were fixed, stained with F77 (green) and CD44 (red), and visualized with confocal microscopy.

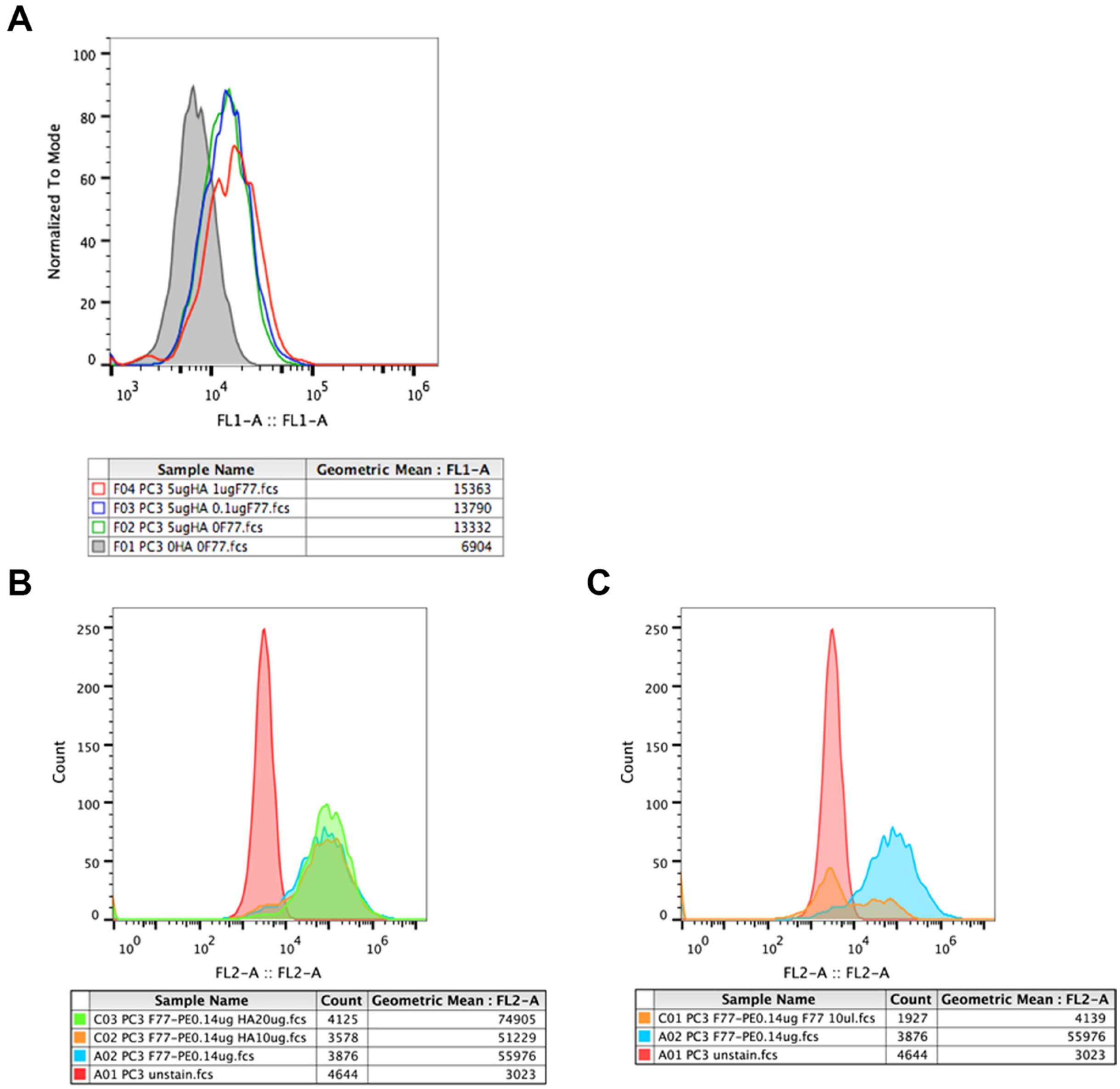

Supplementary Figure 3: F77 does not compete with hyaluronic acid (HA) for binding to CD44 . (A) Five microgram of HA-FITC was used to bind CD44 on PC3 cells in the presence of 0, 0.1 µg or 1 µg / sample of F77. PC3 with no HA-FITC and F77 treatment was used as the negative control (grey peak). (**B**) Binding of the PE-labeled mAb F77 (0.14 µg) to PC3 cells (blue peak) in the presence of 10 µg (orange peak) or 20 µg (green peak) of hyaluronic acid (HA). PC3 with no PE-F77 and HA was used as the negative control (red peak). (**C**) Binding of the PE-labeled mAb F77 (0.14 µg) to PC3 cells (blue peak) in the presence of 14 ug unlabeled F77 (orange peak). This was used as a positive control for Supplementary Figure 3B. The result shows that over 100-fold of hyaluronic acid in molar concentration fails to reduce the F77-PE binding signal, suggesting that HA and F77 bind to different regions of CD44 on PC3 cells.

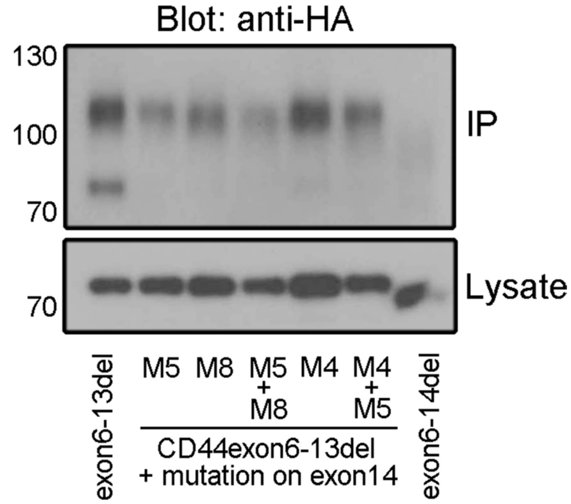

Supplementary Figure 4: Multiple glycosylation sites on CD44v10 contribute to F77 binding. 293T cells were transfected with FUT1 and pIPHA2-CD44exon6-13del plasmid with different O-glycosylation amino acid mutation on exon 14. FUT1 and pIPHA2-CD44exon6-13del was used as the positive control, while FUT1 and pIPHA2-CD44exon6-14del worked as the negative control. CD44 mutants with the removal of glycosylation at single sites (M5, M8, and M4) or multiple sites (M5+M8 and M4+M5) were precipitated by F77 and blotted with the anti-HA-HRP antibody. The lower panel showed the expression of HA-tagged CD44 proteins in cell lysates as the loading control.

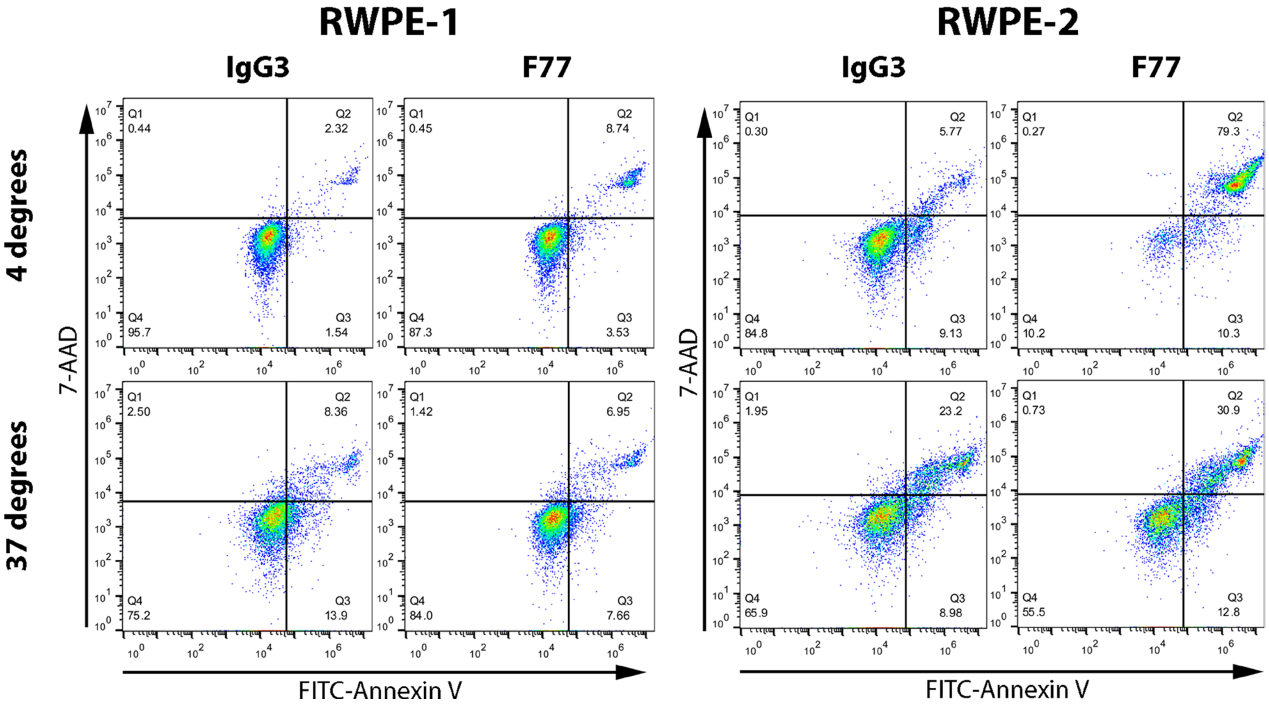

Supplementary Figure 5: F77 induced dramatic apoptosis in RWPE-2 cell at the cold temperature. Left panel: the RWPE-1 cell line (F77 negative); Right panel: the Ki-RAS transformed RWPE-2 cell line (F77 positive). Cells were treated with 10 µg/mL mAb F77 or control mouse IgG3 antibody on ice or in the 37°C water bath for 30 min. After the antibody treatment, cells were washed and stained with the FITC- Annexin V and 7-AAD staining at room temperature for 15 min before the FACS analysis. Antibody treatment did not result in significant apoptosis in RWPE-1 cells at any temperature. Compared with the control IgG3 treatment, F77 treatment resulted in dramatic apoptosis/necrosis (Annexin V positive) in RWPE-2 cells at 4°C (89.6%, Q2 plus Q3 vs 14.9%). At 37°C, F77 only induced cell death modestly (43.7% vs 32.2%, Q2 plus Q3).

Supplementary Table 1: Primers used in this study

Primer name	Primer sequence	Function
CD44_F	CD44-START-FW_ new2: AAAGAATTCTATGGACAAGTTTTGGTGGCA (EcoRI)	To construct pIPHA2-CD44 full length vector
CD44_R	CD44-STOP-RV: AAACTCGAGTTACACCCCAATCTTCATGTC (XhoI)	
CD44exon6-13del_F	CACAGACAGAATCCCTGCTACCAGGAATGATGTCACAGGTGGAAG	To generate CD44 exon6-13 deletion mutant from pIPHA2-CD44 vector
CD44exon6-13 del _R	CTTCCACCTGTGACATCATTCCTGGTAGCAGGGATTCTGTCTGTG	
CD44exon6-14 del _F	GCACAGACAGAATCCCTGCTACCGACCAAGACACATTCCACCCCAGTG	To generate CD44 exon6-14 deletion mutant from pIPHA2-CD44 vector
CD44exon6-14 del _R	CACTGGGGTGGAATGTGTCTTGGTCGGTAGCAGGGATTCTGTCTGTGC	
CD44exon14M1_F	CCTGCTACCAGGAATGATGTCGCCGGTGGAAGAAGAGACCC	To generate glycosylation site mutation M1 on exon 14 based on pIPHA2-CD44exon6-13del vector
CD44exon14M1_R	GGGTCTCTTCTTCCACCGGCGACATCATTCCTGGTAGCAGG	
CD44exon14M2_F	GAAGAGACCCAAATCATGCCGAAGGCTCAACTAC	To generate glycosylation site mutation M2 on exon 14 based on pIPHA2-CD44exon6-13del vector
CD44exon14M2_R	GTAGTTGAGCCTTCGGCATGATTTGGGTCTCTTC	
CD44exon14M3_F	CATTCTGAAGGCGCCGCTACTTTACTGGAAGG	To generate glycosylation site mutation M3 on exon 14 based on pIPHA2-CD44exon6-13del vector
CD44exon14M3_R	CCTTCCAGTAAAGTAGCGGCGCCTTCAGAATG	
CD44exon14M4_F	CTACTTTACTGGAAGGTTTCACCTCTCATTACCCACAC	To generate glycosylation site mutation M4 on exon 14 based on pIPHA2-CD44exon6-13del vector
CD44exon14M4_R	GTGTGGGTAATGAGAGGTGAAACCTTCCAGTAAAGTAG	
CD44exon14M5_F	CTTTACTGGAAGGTTATGCCGCTCATTACCCACACACG	To generate glycosylation site mutation M5 on exon 14 based on pIPHA2-CD44exon6-13del vector
CD44exon14M5_R	CGTGTGTGGGTAATGAGCGGCATAACCTTCCAGTAAAG	
CD44exon14M6_F	GGTTATACCTCTCATTTCCCACACGCCAAGGAAAGCAGGACC	To generate glycosylation site mutation M6 on exon 14 based on pIPHA2-CD44exon6-13del vector
CD44exon14M6_R	GGTCCTGCTTTCCTTGGCGTGTGGGAAATGAGAGGTATAACC	
CD44exon14M7_F	CCACACACGAAGGAAGCCAGGGCTTTCATCCCAGTG	To generate glycosylation site mutation M7 on exon 14 based on pIPHA2-CD44exon6-13del vector
CD44exon14M7_R	CACTGGGATGAAAGCCCTGGCTTCCTTCGTGTGTGG	
CD44exon14M8_F	CCTTCATCCCAGTGGCCGCTGCTAAGGCCGGGTCCTTTGGAG	To generate glycosylation site mutation M8 on exon 14 based on pIPHA2-CD44exon6-13del vector
CD44exon14M8_R	CTCCAAAGGACCCGGCCTTAGCAGCGGCCACTGGGATGAAGG	
CD44exon14M9_F	CCTCAGCTAAGACTGGGGCCTTTGGAGTTACTGC	To generate glycosylation site mutation M9 on exon 14 based on pIPHA2-CD44exon6-13del vector
CD44exon14M9_R	GCAGTAACTCCAAAGGCCCCAGTCTTAGCTGAGG	
CD44exon14M10_F	GGGTCCTTTGGAGTTGCCGCAGTTGCCGTTGGAGATTCC	To generate glycosylation site mutation M10 on exon 14 based on pIPHA2-CD44exon6-13del vector
CD44exon14M10_R	GGAATCTCCAACGGCAACTGCGGCAACTCCAAAGGACCC	
CD44exon14M11_F	GCAGTTACTGTTGGAGATGCCAACGCTAATGTCAATCGTTCC	To generate glycosylation site mutation M11 on exon 14 based on pIPHA2-CD44exon6-13del vector
CD44exon14M11_R	GGAACGATTGACATTAGCGTTGGCATCTCCAACAGTAACTGC	
CD44exon14M12_F	CTCTAATGTCAATCGTGCCTTAGCTGGAGACCAAGACAC	To generate glycosylation site mutation M12 on exon 14 based on pIPHA2-CD44exon6-13del vector
CD44exon14M12_R	GTGTCTTGGTCTCCAGCTAAGGCACGATTGACATTAGAG	
CD44exon14M4M5_F	GGCTCAACTACTTTACTGGAAGGTTTCGCCGCTCATTACCCACACACGAAGGAAAGC	To generate glycosylation site mutation M4 and M5 on exon 14 based on pIPHA2-CD44exon6-13del vector
CD44exon14M4M5_R	GCTTTCCTTCGTGTGTGGGTAATGAGCGGCGAAACCTTCCAGTAAAGTAGTTGAGCC	
PC3Fut1KOcheck_F	TGGCCTTCCTGCTAGTCTGT	To confirm CRISPR knockdown of Fut1 in PC3 cell line
PC3Fut1KOcheck_R	TGGCATACTGTCCCATCTGA	
